# Consumer and community involvement in health and medical research: evaluation by online survey of Australian training workshops for researchers

**DOI:** 10.1186/s40900-016-0030-2

**Published:** 2016-05-09

**Authors:** Anne McKenzie, Kirsten Alpers, Jane Heyworth, Cindy Phuong, Bec Hanley

**Affiliations:** 1 Consumer and Community Involvement Program, The University of Western Australia School of Population Health and the Telethon Kids Institute, 35 Stirling Highway, Crawley, WA 6009 Australia; 2 TwoCan Associates, 59 Wickham Hill Hurstpierpoint, West Sussex, BN6 9NR UK; 3 School of Population Health, The University of Western Australia, 35 Stirling Highway, Crawley, WA 6009 Australia

**Keywords:** Consumer and community involvement, Consumer and community participation, Patient and public involvement, Training, Awareness, Attitudes

## Abstract

**Plain English Summary:**

In Australia, since 2009, the Consumer and Community Involvement Program (formerly the Consumer and Community Participation Program) has developed and run workshops to help people working in health and medical research involve more consumers (patients) and community members (the public) in their research. In 2012, workshop attendees were invited to do an online survey to find out the effect, if any, that attending a workshop had on their awareness of and attitudes to consumer and community involvement. They were also asked about changes in their behaviour when it came to the involvement of consumers and the community in their work. The study found that, for people who answered the survey, more than double the number found consumer and community involvement very relevant after attending a workshop, compared with the number who thought that before attending one. Also, amongst those who answered the survey, 94 % thought that the workshop increased their understanding about involvement.

**Abstract:**

**Background**

There is limited evidence of the benefits of providing training workshops for researchers on how to involve consumers (patients) and the community (public) in health and medical research. Australian training workshops were evaluated to contribute to the evidence base. The key objective was to evaluate the impact of the workshops in increasing awareness of consumer and community involvement; changing attitudes to future implementation of involvement activities and influencing behaviour in the methods of involvement used. A secondary objective was to use a formal evaluation survey to build on the anecdotal feedback received from researchers about changes in awareness, attitudes and behaviours.

**Methods**

The study used a cross-sectional, online survey of researchers, students, clinicians, administrators and members of non-government organisations who attended Consumer and Community Involvement Program training workshops between 2009 and 2012 to ascertain changes to awareness, attitudes and behaviours related to consumer and community involvement in health and medical research.

**Results**

Changes in awareness and attitudes were demonstrated by more than double the number of respondents finding involvement very relevant after attending a workshop compared with those who did so before attending; 94 % of respondents agreed that the workshops increased their understanding of how involvement can add value to research.

**Conclusions**

The training workshops raised awareness and increased relevance of consumer and community involvement among Australian researchers who attended a workshop and responded to the survey. The results of the survey are also suggestive that the training led to behaviour changes through increased consumer and community involvement.

**Electronic supplementary material:**

The online version of this article (doi:10.1186/s40900-016-0030-2) contains supplementary material, which is available to authorized users.

## Background

### Consumer and community involvement in research in Australia

Consumer and community involvement in health and medical research in Australia has seen a growth in both interest and implementation over the past several decades. In Australia, the term ‘consumer and community involvement’ is used to describe active involvement in decision making about research priorities, policy and practice. In other countries, such as the United Kingdom and the United States, the terms ‘patients’ and the ‘public’ are used in place of ‘consumers’ and ' community’. Early initiatives by Aboriginal communities and the National Health and Medical Research Council (NHMRC) led to greater community involvement in health and medical research and raised awareness of the potential benefits of consumer and community involvement [1]. In 2002 the NHMRC and the Consumers Health Forum of Australia (CHF) released a joint Statement on Consumer and Community Participation in Health and Medical Research [2]. This Statement encouraged involvement of consumers and community members in health and medical research as active partners in the development, design, conduct and dissemination of research.

Despite these national initiatives, uptake of consumer and community involvement in health and medical research in Australia is still ad-hoc. For example, involvement is a requirement of some funding bodies, such as Cancer Australia and the National Breast Cancer Foundation, who assess researchers’ plans for consumer and community involvement as part of their grant review process [3, 4], but not of others, including Australia’s NHMRC.

### Training needs in consumer and community involvement

In the UK, patient and public involvement has been a policy directive in the National Health Service’s research and development process since 1999 [5]. A variety of training options have been offered to researchers to help them to implement this directive. Dudley et al. (2015) have noted there have been few previous empirical studies of patient and public involvement training for researchers. Their qualitative study of patient and public involvement in clinical trials interviewed 31 researchers who had involved patients in their research, of whom 13 had accessed training on involvement [6]. They reported that learning “how to do PPI, what worked for others and how PPI can benefit research” had been useful [6]. Among the 18 researchers interviewed who had not accessed training, nine reported they would value training on “how and when to involve PPI contributors, how to optimise their input, what is expected of PPI contributors, how PPI benefits research and guidance on payment for PPI” [6]. Likewise, the UK National Institute for Health Research (NIHR) University College Hospitals Biomedical Research Centre and UCL Partners [7] found that similar areas were identified as training needs by researchers. Focus groups of lay participants emphasised that training should highlight the value of involving the public and that researchers needed help in improving how they communicate with lay people [7]. Dudley et al. (2015) also found that among the researchers who had not attended training, the main need for involvement that they identified was finding “suitable people” [6]. The nine researchers who reported reluctance to receive training indicated that training was limited by the lack of evidence on which involvement strategies are effective [6].

The impact of a series of training workshops offered in 2014 to researchers connected with the NIHR University College Hospitals Biomedical Research Centre and UCL Partners was evaluated six months after the workshops were delivered, using surveys as well as telephone and face-to-face interviews. This evaluation found that the training had led to increased skills and activities in most (but not all) areas [7]. Understanding of involvement and confidence to implement involvement activities increased from 22 to 86 % and 14 to 54 % immediately after the workshop, respectively. Awareness of the resources available to support involvement rose from 12 to 67 % immediately after the workshop. The six month follow-up indicated that these trends were maintained.

A survey conducted by INVOLVE in 2008 [8] identified that training was a priority for researchers in England. INVOLVE subsequently collated information on existing training and support in England and led an NIHR workshop on learning and development for public involvement in research in 2013. The report from this workshop identified the main risk to the increasing level of involvement across the NIHR as being a failure to ensure coordinated and cost-effective learning opportunities [9]. Delegates at the workshop established a working group to plan an NIHR-wide approach to learning and development. The report produced by this working group noted three topics that were considered most pressing: provision of training for researchers and the public; developing collaborative approaches; and sharing good practice for training and support [10].

With an aim of increasing consumer and community involvement in research activities, The University of Western Australia School of Population Health (the School) established a Consumer and Community Involvement Program (the Program) in 1998. The Program (formerly known as the Consumer and Community Participation Program) has been run jointly with the Telethon Kids Institute (the Institute) since 2002. The Program was developed initially in response to community concerns about linked data research and is underpinned by the philosophies of the NHMRC and CHF Statement on Consumer and Community Participation in Health and Medical Research [2]. The Program aims to increase partnerships between consumers, community members and researchers to make decisions about research priorities, policies and practice in Western Australia (WA).

The early focus of the Program was to link researchers, consumers and community members and provide advocacy and advice. In 2004, a Strategic Plan and involvement policies for the School and the Institute were developed. Since 2004, governance, methods of involvement, evidence building and expanded training have become major aspects of the work conducted by the Program at the School, the Institute and other Western Australian and national organisations [11]. Training for researchers in consumer and community involvement is one part of the overall training aspect of the Program, which also includes training for consumers and community members, lectures and seminars. The Program has also developed methods to support researchers to find ‘suitable people’, a need identified by researchers in Australia as well as the UK researchers noted by Dudley et al. (2015) [6]. While the Program works with health and medical researchers and policy makers in Australia, it operates in collaboration with, rather than from within, a scientific research paradigm.

In 2008, the inaugural Involving People in Research National Symposium, organised by the Program, was held in WA. The Symposium advocated for consumer and community involvement in research and was attended by consumers, community members, members of non-government organisations, students, researchers, clinicians, administrators and representatives from funding bodies.

All Symposium attendees took part in a workshop to determine barriers to implementing consumer and community involvement and what was needed to move forward. A lack of training was identified as one of four key issues and was discussed at a post-Symposium meeting with the NHMRC in 2008.

Following the Symposium, in 2008 Saunders et al. surveyed research and funding organisations in Australia to identify prevalence, challenges, enabling factors and approaches to consumer involvement in health and medical research [12]. Guidelines and tools to support involvement, evidence of benefit of involvement and effective staff training were perceived as the three main facilitators of consumer and community involvement. However, there were limited data on the effectiveness of training in increasing awareness and practice. In response to the identified need for training the Program deveoped and piloted training workshops for researchers.

Anecdotal feedback and increased requests for support from researchers showed a positive response to the training provided by the Program. In order to build on the indicated changes to awareness, attitudes and behaviour, a formal evaluation of the training workshops was undertaken. It was intended that the results of the evaluation would be shared with research managers and leaders about the benefits of training. It was also considered to be important, as part of the Program’s continuous improvement activities to examine these potential changes and modify the training, if necessary, to maximise the benefits.

Thus we undertook a post-hoc evaluation with the aim of evaluating the effectiveness of researcher training workshops developed by the Program in:

- increasing awareness of the benefits of consumer and community participation
- changing attitudes to future implementation of participation activities
- influencing behaviour in the methods of participation used.

## Methods

### The workshops

The training workshops were developed by the Program in collaboration with Bec Hanley of TwoCan Associates in the UK and were based on workshops run in England for the NIHR. Workshops were first piloted in 2009 and a total of twenty three workshops were organised and facilitated by the Program between 2009 and 2012 with an average attendance of seventeen people. Workshops were held in Western Australia, Victoria, South Australia, the Australian Capital Territory and New South Wales.

The researcher training workshop content covers foundations of consumer and community involvement; why it is important; benefits of and barriers to involvement; ethical considerations; and methods of implementation. There are also presentations of good practice examples from researchers and consumers.

Of the twenty three workshops organised by the Program between 2009 and 2012, eleven were chosen for the evaluation. They were organised and facilitated by both AM and BH and adhered to the method developed by AM and BH for a general researcher audience. Minor changes in response to attendee feedback did occur between the earlier and later workshops but the course content, structure and delivery methods were the same at all workshops included in this evaluation. Workshops not included in the survey did not conform to this method in that they were half day or introduction to involvement workshops (*n* = 5), were for a mixed audience (*n* = 1) or were not organised and facilitated by both AM and BH (*n* = 6).

As the Program works on a continuous improvement model, attendees are asked to fill in a feedback form at the conclusion of each workshop. Feedback forms are completed before attendees leave the workshop and are collected, collated and summarised by Program staff. This feedback has shown that over 80 % of attendees anticipated changes to their work practices as a result of attending a workshop.

### Study population

Researchers, students, clinicians, health administrators and members of non-government community organisations who attended one or more of the 11 selected training workshops were contacted in 2012 and asked to complete the survey. The workshop attendees were contacted through the email address they supplied at the time of the workshop. An information sheet was included with each email and a twofold online consent was sought; consent to take part in the survey and consent to the use of stories/narratives gained in the survey. The survey could only be accessed after consent was submitted. Ethical approval was provided by The University of Western Australia Human Research Ethics Committee (RA/4/1/5125).

### The survey

In this paper ‘consumers’ are current or potential patients or carers and ‘community’ is a group of people sharing a common interest. Whilst the term ‘involvement’ is becoming more widely used in Australia, this was not the case when the survey described in this paper was carried out. Instead the term ‘participation’ was used to include involvement. Therefore the use of ‘participation’ in survey questions and answers is, in this context, synonymous with involvement.

Program staff developed a short, anonymous, online survey in collaboration with researchers from the School and the Institute and BH. The questions covered three areas: awareness of; attitudes to; and behaviours related to consumer and community involvement in the respondent’s research or workplace. The full questionnaire is included in Additional file 1. Piloting of the survey was undertaken by two researchers who were also potential respondents.

Questions relating to awareness included three open-ended questions that asked respondents to identify key messages from the workshop, barriers to involvement and benefits of involvement. Additionally, respondents were asked about the stages of the research cycle in which they involved consumers and community members. The stages included: deciding what to research; deciding how to do it; conducting the research, disseminating the results; and future research directions. Multiple responses could be provided for open-ended questions.

The questions relating to attitude assessed (1) the importance of consumer and community involvement in the respondent’s research/workplace on a six point scale ranging from ‘not necessary’ to ‘very important’; and (2) the relevance to them before and after attending a workshop on a four point scale ranging from ‘not relevant’ to ‘very relevant’. Respondents were also asked to rate the extent to which they agreed that attending the workshop had increased their understanding of how involvement can add value to research. Response options were a 5- point Likert scale ranging from ‘strongly disagree’ to ‘strongly agree’ and respondents were asked to provide reasons/examples.

The survey questions relating to behaviour were asked in order to find out:

- the role consumer and community involvement had in the respondent’s work prior to the workshop
- whether or not the respondent was aware of any workplace procedures to support involvement activities before the workshop
- whether, since attending the workshop, the respondent had been able to or intended to implement involvement activities
- any barriers to implementation identified by the respondent
- whether the respondent had actively increased awareness of consumer and community involvement in their organisation following the workshop

Descriptive analyses included simple frequencies of responses from Survey Monkey. Responses to open-ended questions were categorised by comparing the responses to each question and identifying common themes.

## Results and discussion

Of the 205 attendees at the 11 selected workshops, 180 valid email addresses were obtained. Through these, attendees were contacted and asked if they would participate in the study. One hundred responses were received of whom 60 were researchers, 11 students, six health administrators, five clinicians, five members of non-government organisations or community organisations and 13 other. For the majority of questions, 74 responses were received. No other demographic information was requested in the survey to ensure the anonymity of respondents.

Two of the 100 respondents did not recall the year they attended a workshop. Of the other 98 respondents, 38 participated in 2009, 28 in 2010, 27 in 2011 and 5 in 2012. One respondent who attended training both in 2010 and 2012 was included as a 2012 attendee. The number of training workshops, numbers attending and respondents by year is shown in Table 1.

**Table 1:** Survey respondents by year of attendance at training workshops for researchers

Year of workshop	Number of workshops included in survey	Total participants attending workshops	Number contactable	Number of survey respondents	Respondents as percentage of contactable participants
2009	5	97	79	38	48.1 %
2010	2	41	39	28	71.8 %
2011	3	55	50	27	54.0 %
2012	1	12	12	5	41.6 %
Did not recall				2	
total	11	205	180	100	55.5 %

### Increasing awareness

Key messages from the workshop were identified by 73 respondents. These are summarised in Table 2. Some respondents provided multiple key messages and one respondent indicated that they did not find the workshop useful.

**Table 2:** Key messages, barriers and benefits of involvement reported by survey respondents

Open-ended question	Main theme of response	Number of responses
What were the key messages you took away from the workshop about consumer and community participation?	Value: Perspectives and priorities of consumers and community members provide value	25
	Commitment: Consumers and community members should be involved from the start and at all stages of the research, even if it is challenging	12
	Variation: Participation may occur at various stages of the research cycle; forms and levels of participation can vary	12
	Social justice: Involving consumers and community members is the right thing to do	10
	Quality: Specific benefits to researchers; improvements in the quality of research	10
	Relevance: Participation is relevant to consumers, community members and researchers	8
	Barriers: Acknowledgement that barriers exist	7
	Resources: Resources are available to implement participation	6
	Not useful	1
Have you experienced any barriers to involving consumer and community participation in your research?	No barriers experienced	24
	Resistance: Lack of support for participation from colleagues/organisation	15
	Recruitment and retention: Identifying and recruiting consumers; maintaining communication and participation	12
	Time: constraints on researchers’ time	10
	Resources Financial and other resource constraints	9
	Personal: self-imposed barriers	2
	Not applicable	7
	Yes, but not specified	5
What are the main benefits of implementing consumer and community participation in your research area?	Value: Consumer and community perspectives improve research topics and processes	33
	Relevance: collaboration is relevant to consumers and community members	23
	Translation: impact of results on policy and practice	17
	Perspective: Understanding consumers better and listening to community ‘voice’	12
	Measurable benefits: Improved health outcomes	6
	No benefit/yet to see benefit	6

Most key messages reported by respondents related generally to perspectives and priorities of consumers and community members providing value such as ‘*the value consumer and community members can add to research’* and the ‘*importance of hearing the consumer voice and perspective’*.

Many respondents noted that researchers should ‘*involve consumers from the very beginning’* while others were aware that *‘consumers/community can be involved in various stages of research’* and there are *‘different options for level of consumer involvement’.*

Some respondents mentioned key messages that related to social justice and involvement being the right thing to do, such as *‘consumers should have a voice in research’* and that they *‘have a right to be involved in research that affects them’*.

Other respondents mentioned specific benefits such as *‘increase in recruitment and retention rates*’. That *‘consumers are integral to doing thorough and relevant research’* was mentioned as were barriers and the availability of resources to address them.

Awareness that there is not a one-size-fits-all model of involvement but that *‘engagement with consumers can take many different forms and occur at various stages of the research’* was observed in the comments (*n* = 12), as in the following examples.

*‘there are not only multiple ways of involving consumers and the community, but there are also multiple levels of engagement. Some projects are more suited to one over the other, and not to feel that you can only be all in or all out’*

*‘participation can take many forms and that there are processes for managing this so that participation is constructive and a positive experience for all parties’*

The second open-ended question asked if respondents had experienced any barriers to consumer and community involvement. Twenty four respondents indicated that they had not experienced barriers and seven that the question was not applicable. Of the other 42 respondents to the question, five stated that they had experienced barriers but did not provide details and 37 described barriers that they had experienced. These are summarised in Table 2. Some respondents provided multiple examples.

The *‘additional time and money to undertake the extra meetings and work associated with involving consumers’* was reported by about half of respondents who provided examples. For some respondents *‘finding consumers to participate is difficult’,* particularly in *‘identifying consumers who are not spread very thinly already’*. Other respondents reported that *‘there is resistance from other researchers’* and *‘a lack of organisational support to help implement and support ongoing participation’* while for a minority *‘starting off is a psychological barrier but with initial help and a bit of experience, this will improve’.*

In response to the question about whether the workshop had helped to explore ideas about overcoming barriers, 57 (77 %) of the 74 respondents answered yes and 17 (23 %) answered no. Twenty nine respondents provided comments on how the workshop helped them address barriers. Of these, 24 indicated that they used tools and methods from the training workshop while 5 mentioned networking and collaboration benefits.

The third open-ended question, about the main benefits of implementing consumer and community involvement, was answered by 73 survey respondents and comments are summarised in Table 2. Some respondents listed several benefits. Many comments were general, mentioning that *‘gaining consumer perspective’* will *‘produce better research and advocacy’* as well as *‘suggesting new directions of inquiry’* and having *‘concordance between research and community priorities’.* For some respondents, participation is beneficial as it enables *‘us to do our job properly and make the most impact on the health of the community’.* Understanding and listening to the community ‘voice’ helps in *‘making sure that the research we fund is considered important by the community’* and is closely aligned with comments on *‘ensuring relevance of the research to community/consumer needs’* and *‘implementing and translating research results’.*

Seventy four respondents answered a question on the stages of the research cycle in which they use consumer and community involvement. The most common stages for involvement were in dissemination of results (nearly 80 %) and in conducting the research (nearly 70 %). The proportion involving consumers or the community in deciding what to research and how to do it was around 50 % while over 40 % reported involvement in knowing what to research next. Forty five of the respondents to this question provided examples of how they involve consumers and the community in the different stages of the research cycle. Responses ranged from *‘input at each step of the process’* through recruitment and design of study materials to *‘only experience to date has been the dissemination of research findings’.*

### Changing attitudes

Seventy- four respondents answered the question on the importance of consumer and community involvement in their research/workplace; 90 % considered that it was somewhat (*n* = 27) or very important (*n* = 40). Five respondents considered consumer and community involvement to be slightly important and for two it was of little importance. No respondents answered that involvement was not necessary.

Seventy four respondents answered the question on the relevance of consumer and community involvement to them prior to and since attending the workshop (Table 3). Compared with the level of relevance prior to attending a workshop, more than double the number of respondents found consumer and community involvement very relevant after attending a workshop (*χ* ^2^ = 22.5, *p* < 0.001). The number of respondents who saw limited or no relevance fell from 19 prior to attending a workshop to three after attending a workshop. While three respondents considered consumer and community involvement not relevant prior to attending a workshop, none did so after attending.

**Table 3:** Perception of relevance of involvement prior to and after attendance at workshops

How relevant was consumer and community participation in research to you:	Prior to attending the workshop		Since attending the workshop	
Options	Number of responses	Percentage of respondents	Number of responses	Percentage of respondents
Very relevant	18	24.3 %	42	56.8 %
Relevant	37	50.0 %	29	39.2 %
Limited relevance	16	21.6 %	3	4.0 %
Not relevant	3	4.1 %	0	
total	74	100 %	74	100 %

Respondents were asked if the workshop increased their understanding of how consumer and community involvement can add value to research. There were 74 responses, 70 (94 %) of which agreed (33) or strongly agreed (37) that the workshop increased their understanding. Three gave a neutral response and one disagreed. Respondents were asked to provide reasons for their response and these are summarised in Table 4.

**Table 4:** Respondent reasons for increased understanding of the value of involvement after attendance at workshops

Comments on the impact of the workshop on understanding	Number of comments
Reinforced/increased understanding and awareness	30
Provided tools and strategies to implement involvement	12
Provided evidence and examples from consumers and researchers	9
Provided opportunities to connect with and learn from other attendees	5

Comments included: *‘understanding of the scope and depth of consumer and community participation in research was enhanced significantly following attendance’,* the *‘examples provided were very useful’* and *‘case studies were convincing’.* Some respondents noted the practical aspects of the workshops as they *‘learnt more about methods of involving consumers at all stages of research and received information on resources’* but for a few the benefits came from collaborative opportunities as they *‘learnt from others that had attended’.*

### Influencing behaviour

Seventy four respondents answered the question of what role, if any, consumer and community involvement had in their research or work prior to the workshop. For 28 (38 %), consumers and community members had no role and for 46 (62 %) they had a role as a consumer or community representative on steering committees and reference groups. Respondents were asked if they were aware, prior to the workshop they attended, of procedures at their workplace to support consumer and community involvement. Of the 74 respondents, 49 (66 %) were aware of support and 25 (34 %) were not aware of any support. The types of workplace support that 25 respondents provided are summarised in Table 5.

**Table 5:** Awareness of workplace support for involvement reported by workshop attendees

Workplace support	Number of comments
General awareness of existing support and procedures	8
Engagement with existing consumer and community networks	6
Organisational policy and guidelines in place	5
Presentations on participation conducted at workplace	4
Limited/developing	2

Since attending the workshop, 42 respondents had been able to implement consumer and community involvement in their research or work, 25 intended to, 7 had not implemented any involvement and 26 did not respond. Overall, 90 % of the 74 respondents to this question had implemented or intended to implement involvement after attending a workshop. The types of involvement implemented or intended were elaborated on by 36 respondents (see Table 6).

**Table 6:** Types of involvement implemented, barriers identified and types of awareness-raising reported by respondents since attending workshops

	Number of responses
*Types of participation implemented*	
Consultation with reference/advisory group or forum	13
Engagement with community representative or advocate in planning and research design	7
Involving consumers and community members in general	5
Commenced planning/considering future engagement	4
Sought advice from consumer advocates/other experts	3
Introduced policies/improved awareness in workplace	3
Consumer on study team	1
*Barriers identified*	
Constraints in workplace	4
Too early to determine/intend to implement engagement	4
Time pressures	3
Challenges in research/other priorities	2
Lack of funds	1
*Types of awareness-raising*	
Initiated support or engagement activities	13
Had discussions with or informed colleagues	11
Attended or presented on participation at events	5
Recommended consumer advocates or experts	4
Encountered difficulties in increasing awareness	4
Awareness already existed	4

Fourteen respondents provided comments on what prevented them from implementing involvement and these were categorised (see Table 6). When the respondents were asked if they had actively increased awareness of consumer and community involvement with supervisors, co-workers or their organisation following a training workshop, 55 respondents answered yes (74 % of those who answered the question), 19 answered no and 26 did not respond. Examples provided by 43 respondents who had increased awareness were categorised (see Table 6).

Awareness-raising activities reported included general comments such as *‘it’s an issue we discuss more regularly since our team have attended the workshops’.* Others were more specific about raising awareness ‘*by developing key documents around consumer participation in research and feeding these into the wider organisation’.*

Respondents who mentioned encountering difficulties did so in undefined statements of *‘an ongoing challenge’* and ‘*does not always work’*, but also included *‘I did once mention something at a meeting with interstate colleagues and the silence was a bit deafening, but that makes me even more determined to discuss strategies in the future’* and *‘have suggested additional ways to have consumers/community members involved but this was not seen as important’.*

Through the exchange of knowledge and discussion of the experiences of researchers, consumers and community members, the workshops aimed to raise awareness, encourage more positive attitudes and support behavioural change. Evidence that the workshops influenced awareness and attitudes is indicated by several items. The most common message taken away was the value of consumer and community involvement. The main perceived benefits identified related to value and to relevance. This was supported by the response to the importance of involvement (90 % ‘somewhat important’ or ‘very important’) and by more than double the number of respondents indicating that consumer and community involvement was very relevant after attending a workshop, compared with those who did so before attending one. Only 3 respondents saw limited relevance and none reported that involvement was not relevant after attending a workshop. Ninety four percent of respondents agreed that the workshops increased their understanding of how consumer and community involvement can add value to research. Three key aspects identified as important in improving understanding were: tools and strategies to implement involvement, evidence and examples from the field; and networking.

The results of the survey are suggestive that the training led to behaviour change through increased consumer and community involvement in research or work. Prior to attending a workshop, a third of respondents were not aware of any procedures to support involvement at their workplace. Thirty eight percent of respondents reported that consumers and community members had no role in their research prior to a workshop. Despite this, 90 % of respondents intended to or had been able to implement consumer and community involvement in their research or work since attending a workshop. This is consistent with data on intention to change work practices derived from the post-workshop feedback forms, where over 80 % of attendees anticipated changes to their work practices as a result of attending a workshop.

The respondents identified that the main barriers to consumer and community involvement were resistance from colleagues and the ability to recruit and maintain consumer involvement. A majority of respondents (74 %) had advocated for or promoted consumer and community involvement since the workshop through initiating or supporting involvement activities and through discussions with colleagues.

There are several limitations of this survey. First, it was a post-hoc evaluation that relied on respondents’ self- report of awareness, attitudes and behaviour. While the survey was developed based upon previous impact evaluations, the survey responses are self-reported and the survey has not been evaluated for reliability or validity. However, the survey was conducted partly in response to researchers’ requests that such data were needed to add to the evidence. Second was the varying length of recall between attending training and completing the evaluation survey and the associated reporting bias. Respondents were asked to complete the survey in 2012 and 65 % of respondents had attended training in 2009 or 2010. It is acknowledged that it may be difficult for respondents to distinguish their awareness and attitudes prior to and after the workshops and there may be a tendency to provide socially desirable responses. However, it is expected that recall of involvement activities undertaken as part of the respondents’ work would be more salient events to recall. In addition, changes in behaviour would not necessarily be immediate and time was required to allow for potential implementation of involvement activities. Hence a longer recall allowed us to capture longer term outcomes.

The response proportions were 56 % overall but 41 % (74/180) to the main questions and self-report of behaviours. Some research areas at the School and Institute have expected all their researchers to attend training by the Program, as has been the case for some of the workshops held for external organisations. These organisational expectations have contributed to a diversity of attitudes and experiences among workshop attendees. Potentially the survey respondents were more likely to be those attendees who were supportive of and engaged with consumer and community involvement. However, an evaluation of a UK program [7] found that 51 % of participants self-reported as being involvement enthusiasts at the beginning of the workshop. Dudley et al. (2015) found that nine out of the 31 researchers interviewed did not support the need for training because they had learnt from experience and because they felt that the evidence base need to inform involvement training was lacking. These data suggest that attendees and non-attendees may differ on their views on aspects of training.

In Australia, the researcher training workshops currently are unique to the Program and it is important to develop an evidence base to inform the current and future work of the Program and other agencies. In addition to the survey results and the workshop feedback, the influence of the workshops can been seen in the increase in requests made to the Program for training.

## Conclusions

In conclusion, the results of this survey suggest that the workshops were successful in increasing awareness and supportive attitudes for consumer and community involvement. They are also indicative of increased consumer and community involvement amongst respondents. The results suggest the value of involvement is recognised but the lack of support from organisations, ability to recruit consumers and community members and time constraints were the main barriers. However, the conclusions need to be tempered by the potential for recall bias and other external factors occurring between the workshops and survey that may well have influenced attitudes and behaviour.

Feedback from workshop attendees has also informed development by the Program of a book, entitled Planning for Consumer and Community Participation in Health and Medical Research: A Practical Guide for Health and Medical Researchers to assist researchers in planning and implementing consumer and community involvement [13]. The book is available from the Program’s website [14].

The authors acknowledge that some respondents may also have been influenced by the requirements and policies of the NHMRC and research organisations. Also, the Program has provided a range of resources, practical support, advice and advocacy to researchers before, during and after the development of training workshops. These factors may have influenced the respondents, particularly given the length of time between attending a workshop and completing the survey.

The workshops, initially developed to support interested researchers at the School and Institute, are now recognised by other universities and non-government organisations across Australia, particularly in the area of cancer research, as being effective in supporting the implementation of consumer and community involvement. With the current expansion of the Program, it is hoped that further evaluation will be conducted on the impact of training and other methods of support and advice provided to researchers.

As research funding bodies increasingly expect the inclusion of consumer and community involvement in applications, awareness amongst researchers and research organisations has grown. There is a demand for training from researchers with or without policy directives. Where there was expectation that researchers will involve consumers and the community in their research and training was available, we found increased awareness and understanding and some changes in behaviour. The results of this research project will make a contribution to the limited evidence currently available about the benefits of training for researchers on consumer and community involvement.

## Additional file

Additional file 1: Survey questions for evaluation of Australian training workshops for researchers. (DOCX 491 kb)
