# Supplementary figures and images for: Consumer and community involvement in health and medical research: evaluation by online survey of Australian training workshops for researchers
Source: Res Involv Engagem. 2016 May 9;2:16. doi: 10.1186/s40900-016-0030-2 (PMC5611564; doi:10.1186/s40900-016-0030-2)

Appendix

Survey Page 1


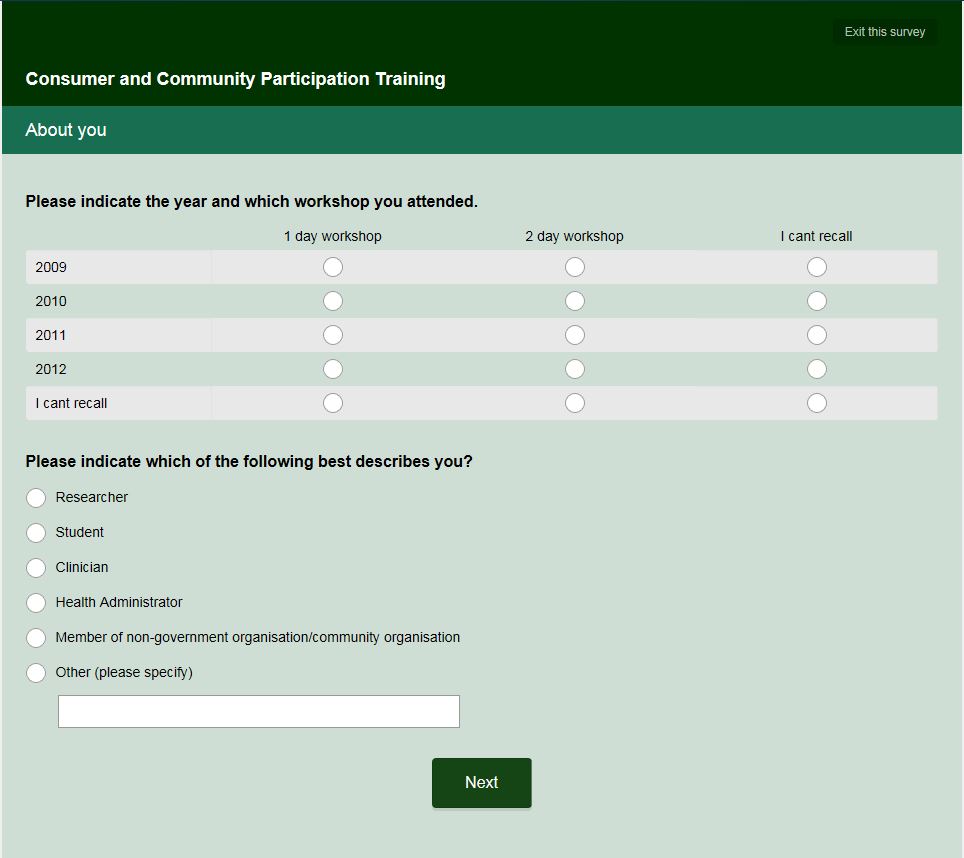


Page 2. Part 1


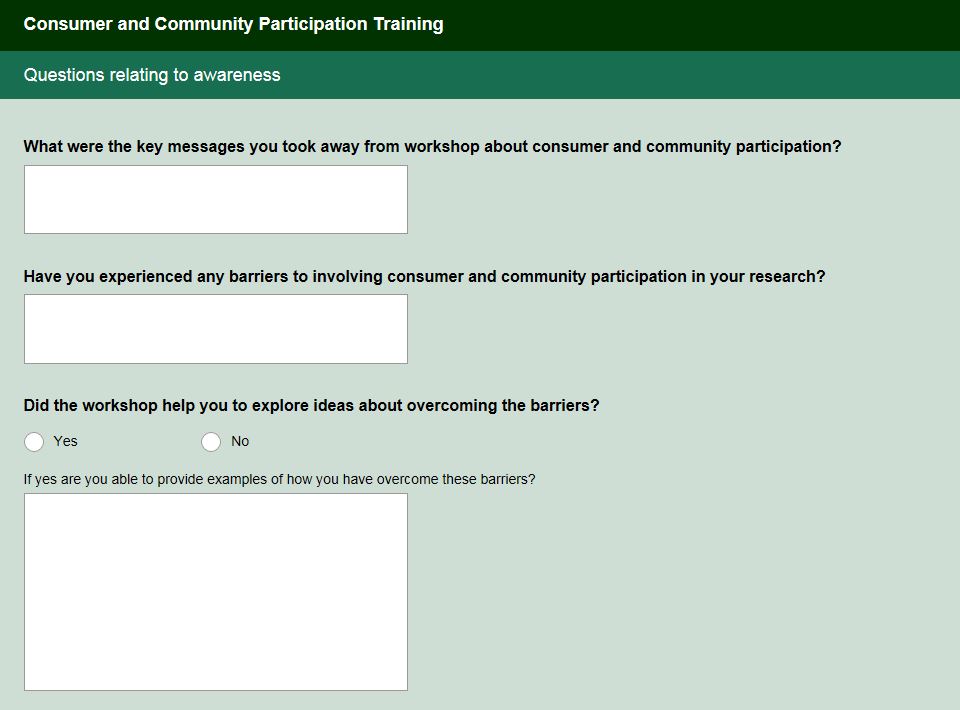


Page 2. Part 2


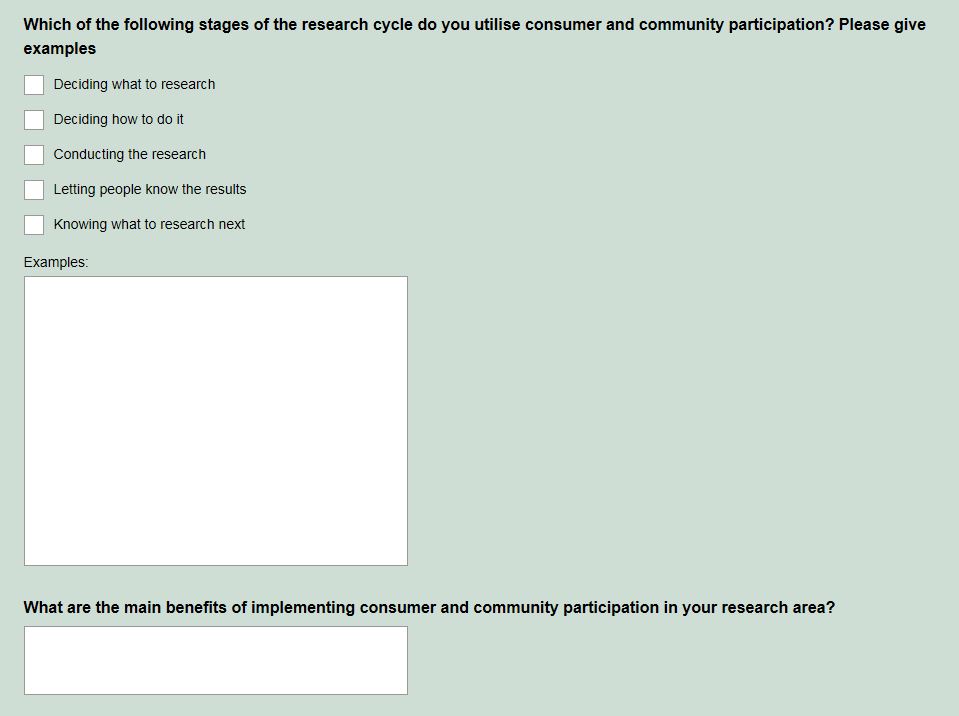


Page 3.


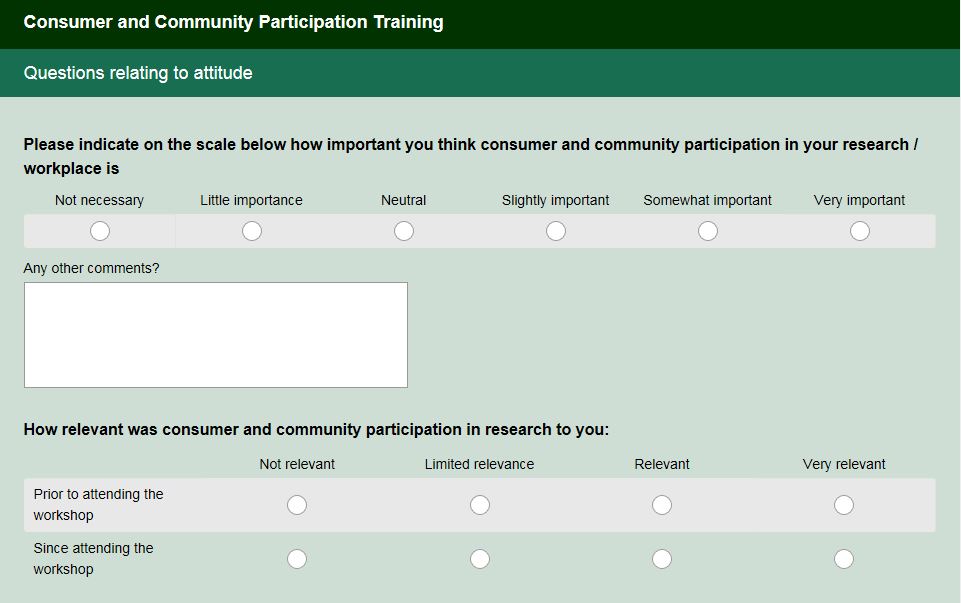


Page 4.


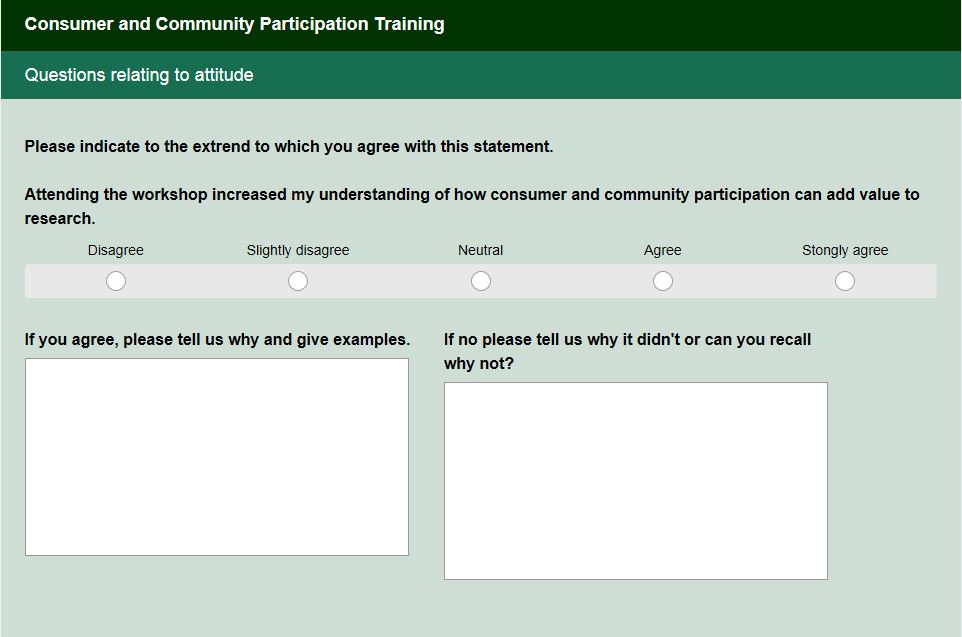


Page 5. Part 1.


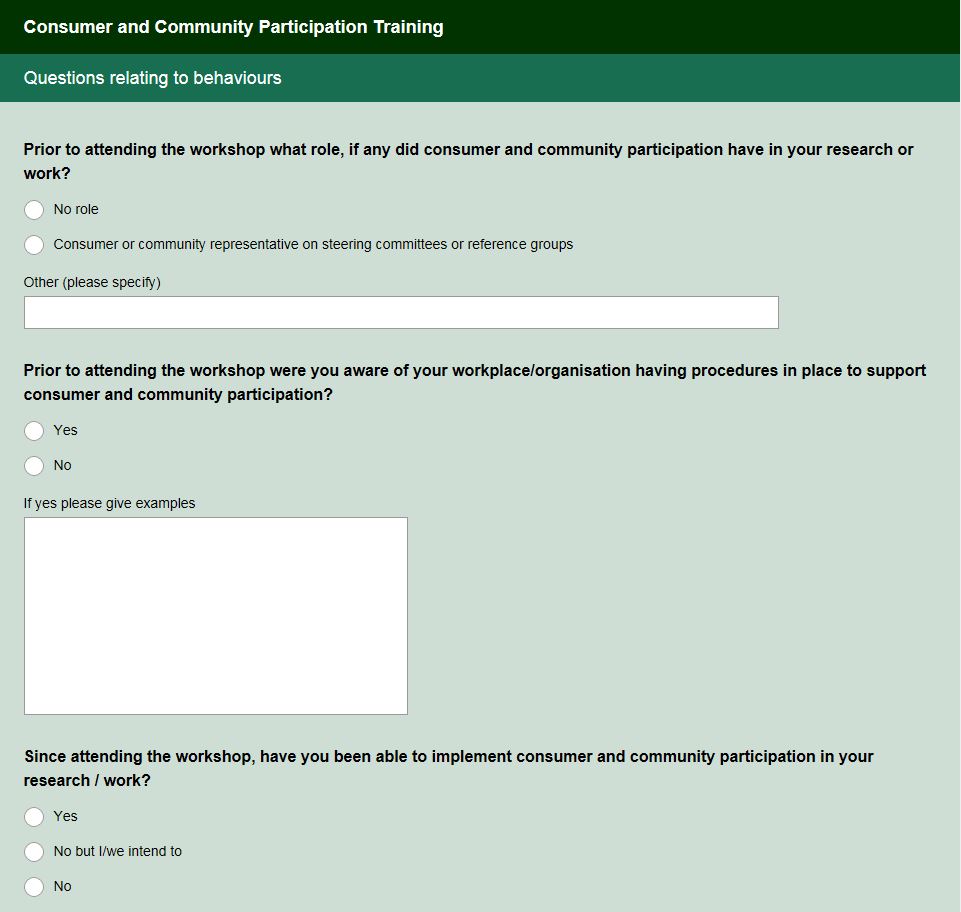


Page 5. Part 2.


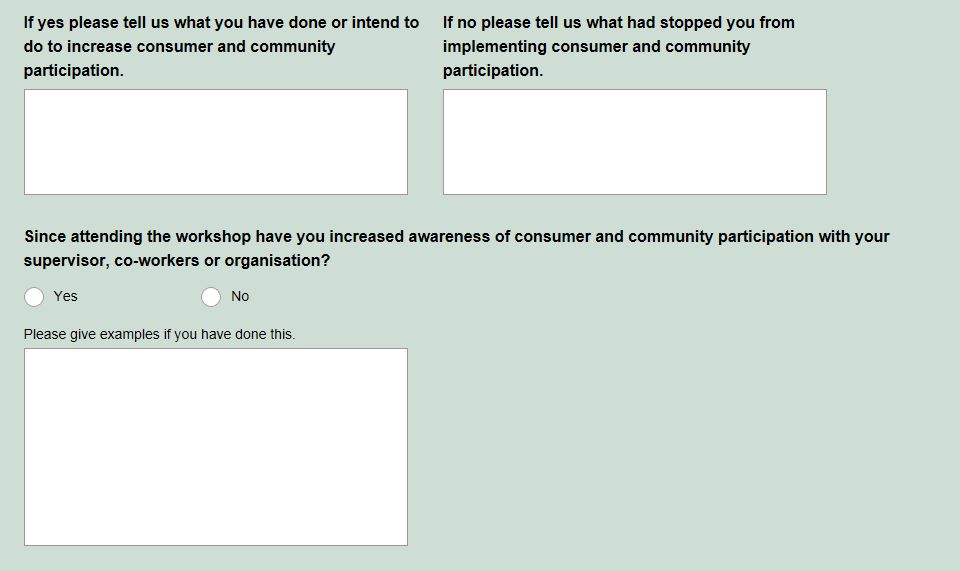


Page 6.


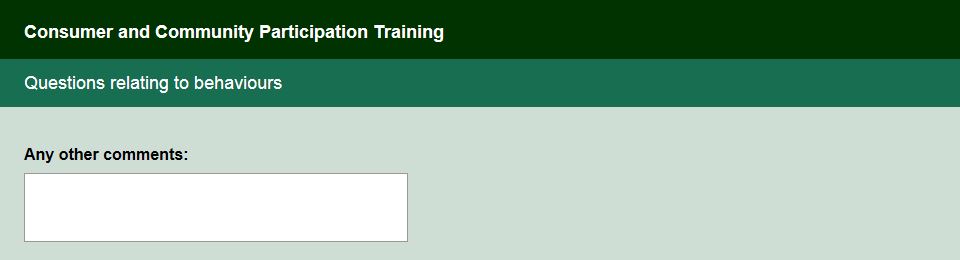


Page 7.


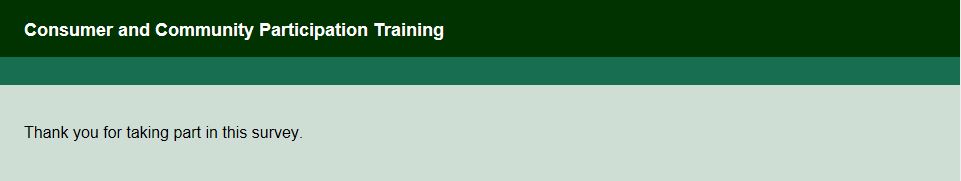

Supplement: Additional file 1: — Survey questions for evaluation of Australian training workshops for researchers. (DOCX 491 kb) [file 40900_2016_30_MOESM1_ESM.docx]
